# Advancing nanolithography: a comprehensive review of materials for local anodic oxidation with AFM

**DOI:** 10.3762/bjnano.17.19

**Published:** 2026-02-09

**Authors:** Matteo Lorenzoni

**Affiliations:** 1 Materials Characterization Facility, Istituto Italiano di Tecnologia, via Morego 30, 16163 Genoa, Italy

**Keywords:** local anodic oxidation, nanofabrication, scanning probe lithography

## Abstract

Local anodic oxidation (LAO), also known as local oxidation nanolithography or oxidation scanning probe lithography has emerged as a versatile technique for nanoscale patterning, leveraging the precision of scanning probe microscopy, relying specifically on atomic force microscopy. This review explores the materials utilized in LAO experiments, including semiconductors, metals, insulators, two-dimensional (2D) materials, and emerging heterostructures. Semiconductors such as silicon and silicon carbide remain foundational due to their controllable oxidation kinetics, while metals like titanium and aluminum offer opportunities for plasmonic and optical applications. 2D materials, including graphene, graphene oxide, and transition metal dichalcogenides, demonstrate unique oxidation behaviors, enabling high-resolution applications in electronics and quantum devices. Recent advancements, such as electrode-free LAO, have expanded the range of applicable materials and improved the precision and scalability of the process. This paper also aims to provide a comprehensive understanding of material selection in LAO and its implications for advancing nanotechnology.

## Review

### 1 Introduction

Advancements in nanotechnology have led to the development of numerous nanoscale fabrication techniques, with scanning probe lithography (SPL) [1] gaining prominence as a versatile method for high-resolution patterning, particularly in the early 2000s. SPL encompasses multiple methods, including nanomechanical processing [2], AFM bias-induced lithography [3], dip-pen nanolithography [4] and thermal scanning probe lithography [5], each catering to unique fabrication requirements. Among the bias-driven techniques, local anodic oxidation (LAO) stands out due to its precise, resist-free process and the compatibility with a wide range of materials [6–9]. LAO uniquely provides maskless patterning under ambient conditions with sub-10 nm lateral resolution, enabling direct oxide formation or material removal with nanoscale precision, capabilities not readily achievable with conventional lithographic approaches. AFM-based LAO originated from earlier STM local oxidation studies [10–12], which first demonstrated tip-induced electrochemical modification of semiconductor surfaces and even early device structures [13]. These STM works established the fundamental mechanism later adopted and expanded by AFM-based approaches. LAO utilizes an AFM to generate localized oxidation patterns through a biased conductive tip. The process relies on the formation of a nanoscale water meniscus [14] at the tip–sample interface, enabling electrochemical reactions that oxidize the substrate, creating protruding nanostructures mainly composed of oxide. Unlike other techniques requiring complex preparation steps, LAO enables direct chemical modification, with precise control of the tip–sample separation during the writing process. This is typically achieved in contact mode, where the tip apex is in direct contact with the substrate, ensuring capillary condensation and the formation of a stable water bridge. However, this approach increases the likelihood of tip wear and plastic deformation of the sharp AFM tip apex. An alternative involves using AFM dynamic mode [15–16], where the electric field induces the formation of the bridge that then stretches and adapts to the tip’s motion. This can be implemented through a dual-pass technique or by controlling the electronic and ionic contributions to the total current [17–18]. While dynamic mode LAO does not eliminate tip wear [19], it significantly reduces its impact.

**Figure 1:**
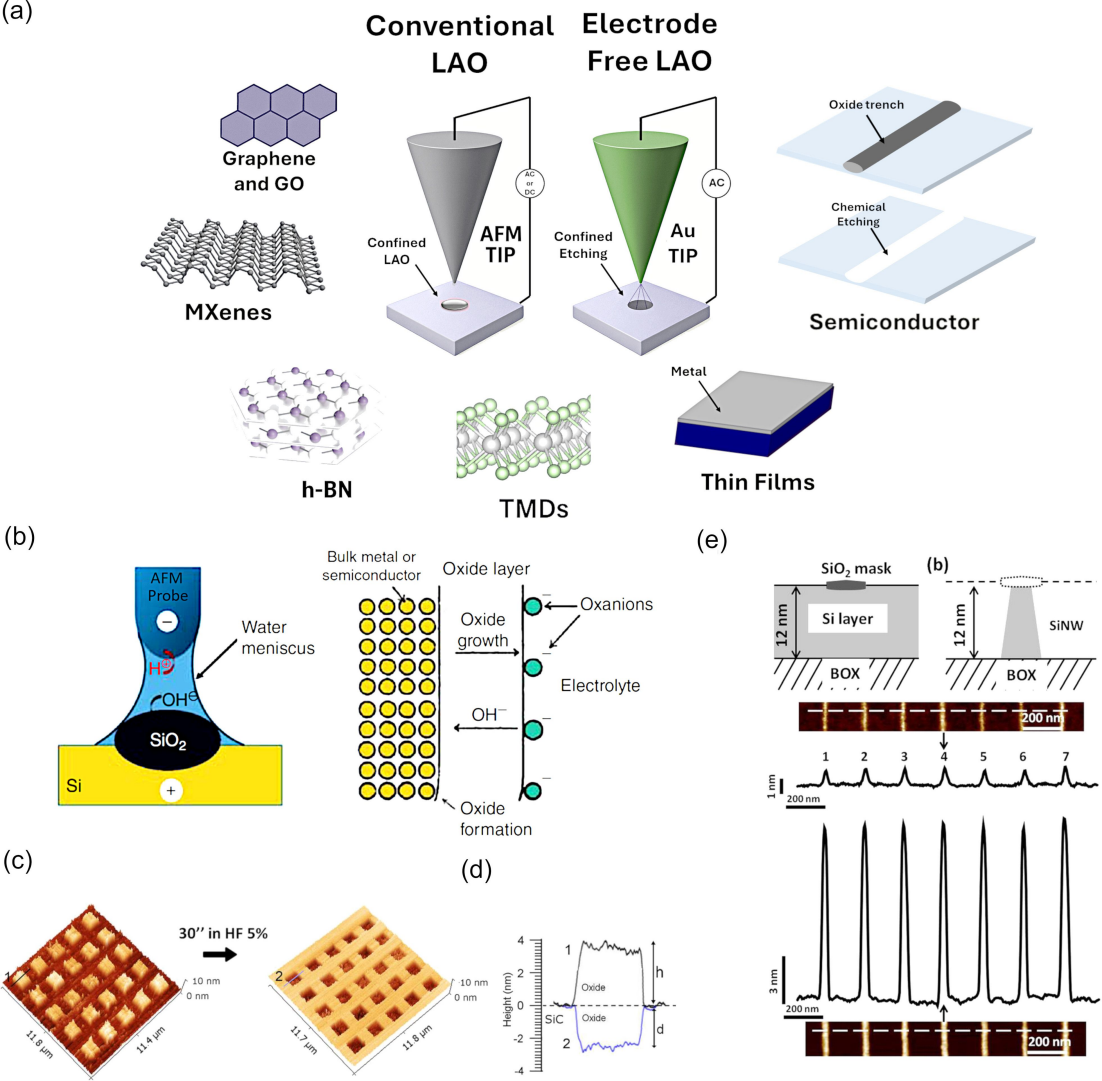
Overview of the local anodic oxidation process and resulting oxide structures. (a) Schematic summary of the main materials addressed in this review and comparison between conventional LAO and electrode-free LAO. (b) Mechanism of oxide growth on a silicon surface during LAO, illustrating the formation of a protruding oxide feature and the corresponding buried oxide volume. Reproduced from [8], M. Lorenzoni and F. Pérez-Murano, “Conductive Atomic Force Microscopy for Nanolithography Based on Local Anodic Oxidation,” in *Conductive Atomic Force Microscopy: Applications in Nanomaterials*, with permission from John Wiley and Sons. Copyright © 2017 Wiley-VCH GmbH. This content is not subject to CC BY 4.0. (c) AFM images of square oxide patterns fabricated by LAO on SiC before and after wet etching. (d) Height profile of the top left corner feature, before (black solid line) and after etching (blue solid line), showing the ratio between protruding and buried oxide. Panels (c and d) adapted from [34], with the permission of AIP Publishing. This content is not subject to CC BY4.0. (e) Schematic of the fabrication process together with AFM topography and cross-sectional views of an array of oxide masks (top) and their corresponding etched silicon nanowires (bottom). Panel (e) adapted from [42], with the permission of AIP Publishing. This content is not subject to CC BY4.0.

As new materials continue to emerge in the field of nanotechnology, this review also aims to highlight the capability of LAO to pattern two-dimensional (2D) materials such as graphene [20–22], hexagonal boron nitride [23] (hBN), transition metal dichalcogenides (TMDs) [21,24–27], metals [18,28–29], as well as traditional semiconductors such as silicon (Si) [6,10,30–31], gallium arsenide (GaAs) [32–33] and silicon carbide (SiC) [34], meaning that SPL has still the potential to revolutionize nanolithography. In the context of 2D materials, LAO facilitates the fabrication of intricate structures such as nanoribbons and transistors, granting proper operation of the fabricated device and preserving the intrinsic material properties [35–36]. For semiconductors, the method allows for precise control over oxide thickness, critical for applications in microelectronics and sensor technology. Oxide thickness can be precisely controlled, enabling the formation of layers as thin as sub-nanometer for SiO_2_ [37] and extending to tens of nanometers for materials such as SiO*_x_*C*_y_* [34], TiO*_x_*, and MoO*_x_* [38]. In contrast, atomic layer deposition (ALD) provides exceptional conformality and atomic-scale thickness control, typically depositing films at 0.1–0.2 nm per cycle [39], making it the preferred technique for large-scale, uniform coatings. However, LAO remains advantageous for high-resolution, direct-write patterning at the nanoscale, despite its limited scalability. Furthermore, the resulting LAO oxide can be easily etched by mild acid solutions [40] or water [24]. A layer of a few nanometers of insulating material (e.g., native oxide, fabricated oxide, or silicon nitride masks [41]) does not represent an obstacle to the LAO process, while bulk insulating materials still represent a major challenge. Figure 1a summarizes the content of this review, showing the materials considered and illustrating a comparison between conventional and electrode-free LAO (EFLAO).

### 2 Principles of local anodic oxidation (LAO)

As already said, LAO is a nanoscale fabrication technique that utilizes an AFM to generate precise oxidation patterns on a substrate. The process is based on the application of a voltage bias between a conductive AFM tip and the sample surface, resulting in localized electrochemical reactions. This section delves into the fundamental principles governing LAO, including its mechanisms, controlling factors, fabrication metrics and advancements in the technique.

#### 2.1 Mechanism of LAO

The key to LAO lies in the formation of a nanoscale water meniscus between the AFM tip and the sample surface, as depicted in Figure 1b. Under ambient humidity, the water layer serves as an electrolyte, enabling electrochemical reactions. When a positive voltage is applied to the AFM tip relative to the substrate, the intense electric field (*E* > 10^7^ V·m^−1^) [23] within the water meniscus drives oxygen-containing ions (e.g., OH^−^ and O^2−^) towards the substrate surface. This triggers the oxidation reaction. At the substrate, the ions react with the material, forming newly grown oxide. Specifically, on Si, LAO-derived oxide exhibits a density of 2.05 g·cm^−3^, lower than the 2.27 g·cm^−3^ density of thermally oxidized silicon [43]. In the case of Si surfaces the chemical reaction leading to SiO_2_ formation is:




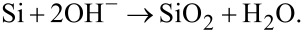




In the case of SiC, carbon atoms are eliminated in the form of carbon dioxide:




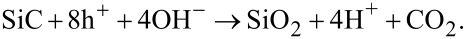




The oxides fabricated on MoX_2_ materials (MoS_2_, MoSe_2_, and MoTe_2_) are instead soluble in water, indicating that the product of the reaction is MoO_3_ [21]:




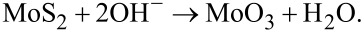




**Figure 2:**
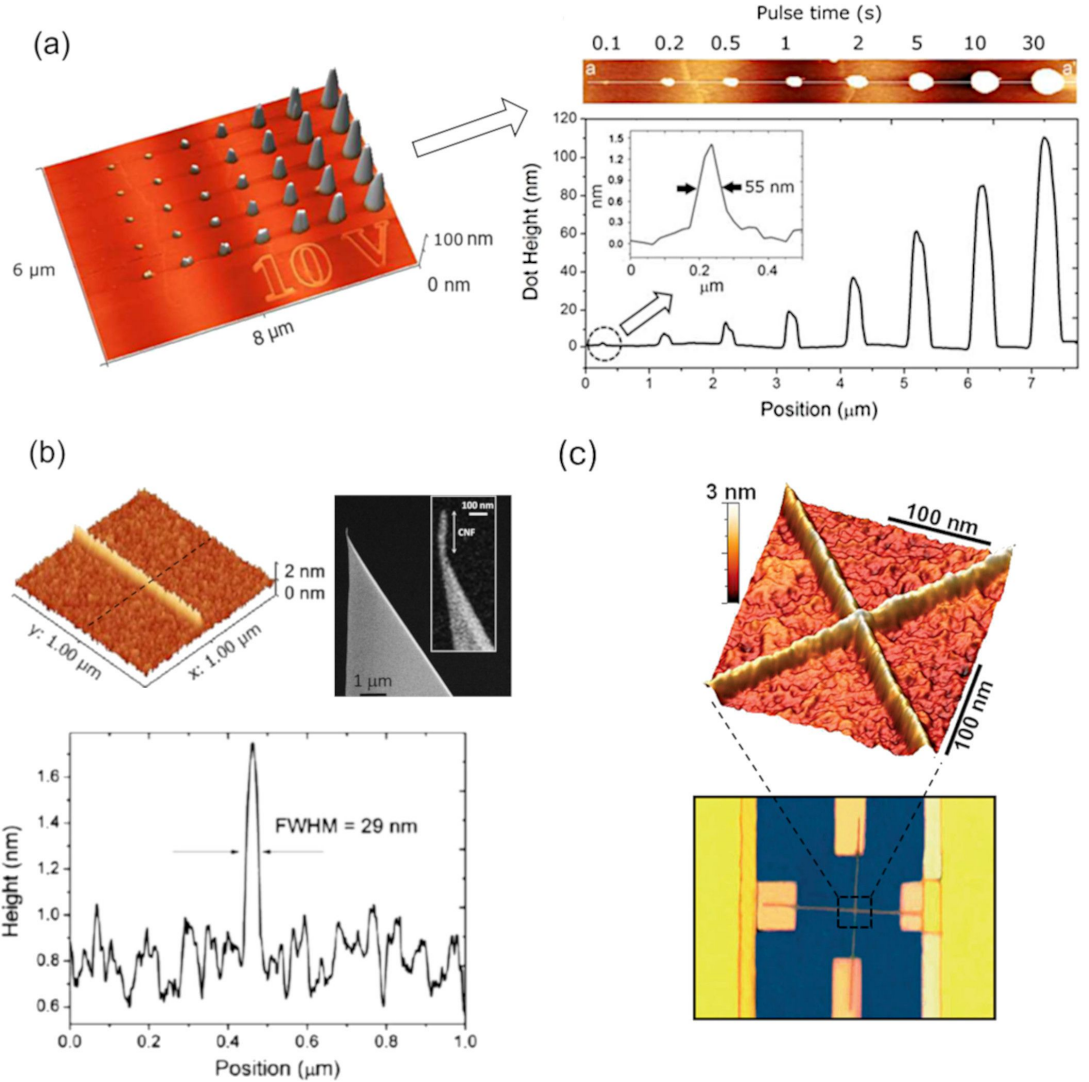
Examples of oxide nanostructures fabricated by local anodic oxidation. (a) AFM topography of an array of single oxide dots produced on a 6H-SiC surface at 40% relative humidity using voltage pulses from 0.1 to 30 s at 10 V. Adapted from [34], with the permission of AIP Publishing. This content is not subject to CC BY4.0. (b) Cross-sectional profile and AFM topography of a single silicon oxide line patterned using a carbon nanofiber (CNF)-functionalized AFM tip. Adapted from [49] (“Boosting the local anodic oxidation of silicon through carbon nanofiber atomic force microscopy probes”, © 2015 G. Rius et al., published by Beilstein-Institut, distributed under the terms of the Creative Commons Attribution 2.0 Generic License, https://creativecommons.org/licenses/by/2.0). (c) Multilevel silicon nanowire transistor fabricated through sequential LAO steps. AFM topography after etching and corresponding device micrograph. Adapted with permission from [50], Copyright 2008 American Chemical Society. This content is not subject to CC BY 4.0.

Similarly, LAO on WSe_2_ produces WO_3_ features that have been etched in water [24]. If we consider semiconductors and metals, most authors agree with an oxidation kinetic consistent with the Cabrera–Mott model [44–45]. As an electrochemical process, LAO is inherently current-dependent. Early studies showed that oxide growth evolves from an initial electronic tunneling regime to ionic transport as the film develops [46]. Dagata and coworkers further demonstrated that charge accumulation within the growing oxide leads to space-charge-limited behavior, reinforcing the self-limiting character of LAO [47]. As the oxide thickens, the local electric field weakens, the supply of reactive species at the water–oxide interface decreases, and stress and trapped charges hinder further ion transport. Together, these effects progressively slow oxidation and ultimately halt growth [48]. This explains why local oxidation experiments on Si report maximum features below 10 nm, while oxide features grown on SiC (with different stoichiometry and density) can reach up to 100 nm height [34]. The protruding oxide observed by AFM represents only part of the total oxide volume. Because oxidation proceeds into the substrate and the formed oxide is less dense than the parent material, a comparable portion (typically 40–60%) develops beneath the surface. This buried component is a general feature of LAO and must be considered when interpreting feature height and oxide thickness [10,48]. This is illustrated in Figure 1c,d, taking into consideration LAO experiments on SiC (Figure 2a).

Beyond the chosen substrate, the thickness and lateral dimensions of the oxide features are governed mainly by the applied voltage, pulse duration, and tip movement speed. This controlled oxidation process is highly effective for fabricating high-resolution patterns, with numerous examples demonstrating the successful creation of silicon oxide nanostructures featuring lateral dimensions well below 100 nm and heights ranging from 1 to 10 nm. The electrochemical reaction in LAO progresses relatively slowly with the water bridge forming rapidly (≈10 µs), but it is the subsequent growth process that may last several seconds. This kind of patterning capability is widespread, and most commercial AFM nowadays implement some interface enabling LAO lithography. Often, the oxide features are used as masks to amplify the pattern depth, as shown in Figure 1e, where silicon nanowires (SiNWs) are created by patterning a SOI wafer [42]. In Figure 2b,c, we show some outcome of LAO experiments aimed to fabricate narrow oxide lines on Si. In one case (Figure 2b), the aspect ratio was enhanced by using a carbon nanofiber (CNF)-functionalized AFM tip [51]; Figure 2c illustrates the scale of a multilevel SiNW transistor, serving as proof that LAO is compatible with performing multiple oxidation steps at the same location during device fabrication [52].

**Figure 3:**
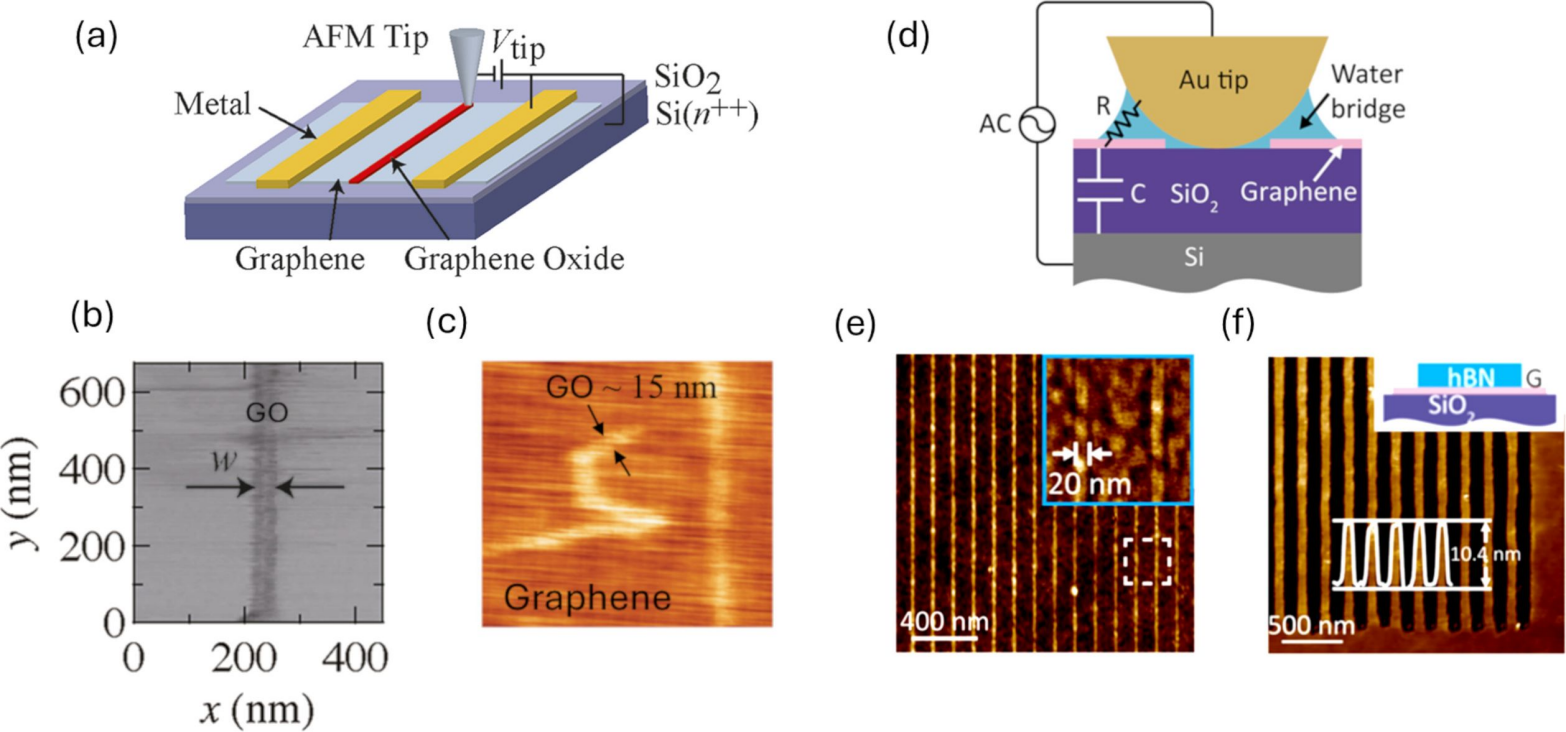
Graphene processing by LAO and electrode-free LAO (EFLAO). (a) Schematic representation of the LAO process on a single-layer graphene flake contacted through top electrodes. (b) Friction-AFM image and (c) topography of graphene oxide features produced by conventional LAO. Panels (a,b) adapted with permission from [22], Copyright 2011 American Chemical Society. This content is not subject to CC BY 4.0. Panel (c) was reprinted from [35], with the permission of AIP Publishing. This content is not subject to CC BY 4.0. (d) Schematic of the electrode-free LAO (EFLAO) configuration, which relies on capacitive coupling and does not require top electrodes. (e) AFM topography of an ultrathin graphene nanoribbon (≈10 nm width) fabricated by EFLAO. (f) AFM topography of an hBN trench patterned using EFLAO. Panels (d–f) adapted with permission from [23], Copyright 2018 American Chemical Society. This content is not subject to CC BY 4.0.

The common experimental setup of LAO on 2D materials flakes is depicted in Figure 3a, where top electrodes are necessary due to the insulating SiO_2_ layer. The nanometer sized GO features obtained are shown in Figure 3b,c. The description of the fabrication steps to enhance the aspect ratio of such features is out of the scope of this work. But it is worth mentioning that most oxides exhibit good selectivity against many common etchants used for dry etching, including reactive ion etching with fluorine- or chlorine-based plasmas, allowing for precise pattern transfer [50,52].

The use of a single probe remains a significant limitation for all SPL methods. In LAO experiments, scanning probes mounted on commercial AFMs typically operate at processing speeds of tens of micrometers per second, restricting the ability to create fast patterns over large areas. Unfortunately, the current state of the art for oxidation scanning probe lithography in terms of subsequent of resolution and throughput has not changed significantly in the last years [53]. The lateral resolution of modified features is usually set around tens on nanometers, with remarkable exceptions of single narrow lines reaching sub-10 nm full width at half maximum [21,50] and machined gaps on 2D materials as narrow as 20 nm [21,23]. The typical throughput of LAO is around a few square millimeters per hour [53]. In comparison, thermal scanning probe lithography (t-SPL), the fastest SPL technique, can reach writing speeds of up to 1 mm·s^−1^, enabling patterning rates of the order of 10–100 mm^2^·h^−1^ under optimal conditions [5]. This puts it in a similar throughput range to electron beam lithography (EBL), which, depending on beam current and pattern complexity, typically achieves 1–100 mm^2^·h^−1^ for high-resolution writing [54–55]. Table 1 incorporates t-SPL, field-emission lithography (FEL), and EBL to provide reference benchmarks against which the capabilities of LAO can be evaluated. t-SPL is the fastest tip-based method, using a heated cantilever to induce thermochemical changes in an ultrathin resist, and is therefore an important comparator for writing speed and patterning rate. FEL, based on the emission of low-energy electrons from an AFM tip, provides sub-10 nm resolution in ultrathin molecular resists and thus represents the upper limit of tip-based spatial precision. EBL, although not a scanning probe technique, remains the industrial standard for high-resolution patterning and sets the baseline for assessing scalability and throughput. Their inclusion highlights where LAO stands relative to established nanolithography methods and clarifies its unique advantages and limitations.

**Table 1:** Comparison of metrics: LAO vs t-SPL, FEL and EBL.^a^

Metric	Local anodic oxidation (LAO)	Thermal scanning probe lithography (t-SPL)	Field-emission lithography (FEL)	Electron beam lithography (EBL)

throughput	1–10 µm^2^·h^−1^	10–100 mm^2^·h^−1^ [5]	1–10 µm^2^·h^−1^	10–100 mm^2^·h^−1^ [5]
writing speed	micrometers per second to tens micrometers per second	up to 1 mm/s [5]	similar to EBL: nanometers per second to micrometers per second	tens of millimeters per second (limited by dose requirements)
etch selectivity	moderate (thin oxide mask, limited selectivity)	moderate (thin thermal resist layer)	high (depends on resist; PMMA commonly used)	high (i.e., hydrogen silsesquioxane (HSQ) and PMMA)
aspect ratio	low; typically, <2:1 due to shallow oxide features	moderate; aspect ratios up to 4:1 achievable with optimized processes	similar to EBL	high; aspect ratios exceeding 10:1 possible with appropriate resists and etching techniques
resist/mask material	native oxide formed (e.g., SiO*_x_* and TiO*_x_*)	thermal resist (no development needed)	PMMA, HSQ, and calixarene [56]	wide selection (e.g., PMMA and HSQ)
scalability	low (serial tip-based, though arrayed LAO emerging)	moderate (parallel tip arrays under development)	low (self-actuated tip array is possible)	moderate to high (especially with multibeam systems)
resolution	<10 nm [50]	10–20 nm (lateral) [5]; sub-10 nm possible; single-digit nanometer resolution demonstrated [57]	<10 nm (industry standard)	–

^a^Where not explicitly stated, the quantitative data have been derived from sources included in the reference list.

#### 2.2 Factors influencing oxidation

The effectiveness and precision of LAO depend strongly on ambient humidity, which governs formation and size of the water meniscus. Optimal relative humidity (RH) between 35% and 55% ensures reproducible oxidation, while higher humidity enlarges the meniscus and broadens oxide lines. Conversely, lower humidity reduces meniscus volume and enables narrower, better-defined features. Although RH of 35–55% is widely used for Si and other common substrates, the optimal range can shift depending on the substrate surface chemistry, hydrophilicity, and the tip material or coating. Studies such as Garcia et al. [15] and Snow et al. [58] systematically demonstrate this dependence, showing a strong correlation between humidity control and achievable resolution in LAO. Voltage and pulse time also play an important role, when the voltage pulse applied is fixed (i.e., tens of milliseconds) oxide size increases linearly with pulse amplitude but logarithmically with pulse width [59]. As expected, higher voltages and longer oxidation times increase oxide thickness but may lead to tip wear and reduced lateral resolution. During LAO, the tip morphologically and chemically degrades; therefore, the choice of tip material (e.g., n^+^-doped silicon, metal coating, or solid alloy) affects oxidation efficiency, with solid tips performing better than coated tips in terms of durability. Tip shape and sharpness play also a crucial role as a main regulator of minimum feature size. Carbon nanotube (CNT)-functionalized AFM tips have been explored for LAO [49,60–61] because their high aspect ratio and good conductivity can, in principle, enable sharper and more controlled oxidation. However, their practical performance is limited by mechanical fragility and variability in CNT integration. For this reason, CNT tips may show excellent results in specific cases [62] but do not consistently outperform robust commercial Si tips. Conductive or semiconductive substrates facilitate the process, while insulating substrates may require additional surface treatments [63] or modified LAO setups. Topography also plays a significant role: While low-roughness surfaces facilitate the process, RMS roughness above roughly 1–2 nm can distort the meniscus and induce lateral broadening of the oxide features. Notably, Ryu et al. demonstrated that oxygen plasma pretreatment of MoS_2_ flakes can spatially confine the oxidation process, enabling the formation of well-controlled dot-shaped features [21].

#### 2.3 Advanced techniques

Recent advancements have expanded the capabilities of LAO to the electrode-free EFLAO [23]. This technique employs high-frequency AC fields to induce oxidation, removing the need for contacted electrodes and broadening the range of usable substrates as shown in Figure 3d. It is important to notice that this technique can be labeled as an anodic oxidation etching. Using AC rather than DC voltage for local anodic oxidation through capacitive coupling, it can be applied to any sample, even those without electrodes or electrical contacts. This method simplifies sample preparation, broadens the range of compatible materials, and enhances the reliability and quality of lithographic cuts, all without the need for additional AFM equipment. If applied to graphene or boron nitride, authors of [23] have shown how reaction by-products are efficiently removed, escaping as gas (i.e., CO_2_ in the case of graphene). The resulting patterns exhibit excellent quality with minimal defects (Figure 3e,f). However, a potential drawback is the use of Au-coated tips under contact-mode conditions with applied forces around 1500 nN, which may lead to tip coating wear if the ohmic contact is not facilitated by the presence of a water meniscus. Despite this, the authors demonstrate consistent and reproducible patterning over hundreds of micrometers, achieved at writing speeds typical for LAO experiments.

In a recent study, Shilov et al. [64] proposed a similar approach to EFLAO, demonstrating efficient patterning on multilayer heterostructures composed of graphene, graphite, hexagonal boron nitride (hBN), NbSe_2_, and WSe_2_. Their method leverages the enhancement of the electric field, achieved by assembling the heterostructures on conductive graphite flakes. They suggest that the mechanism of electrical breakdown may play a more significant role than previously assumed, potentially overshadowing the electrochemical reactions traditionally attributed to the process. Although field-induced breakdown may contribute in some electrode-free geometries, current evidence does not rule out a significant anodic oxidation component; therefore, the term EFLAO is retained to describe processes in which electrochemical oxidation remains operative alongside possible breakdown-assisted effects.

For comparison, it is worth mentioning the non-oxidative SPL technique FEL [57], which has recently proven its efficiency and reliability. It exploits a Fowler–Nordheim-type emission of low-energy electrons from a conductive AFM tip. This beam of low-energy electrons can induce highly confined positive and negative tone as well as self-development reactions within ultrathin molecular resist layers [56,65–66], with resolutions reaching sub-10 nm scales [67]. As demonstrated by Prof. Ivo W. Rangelov and colleagues, FEL relies on the high-intensity electric fields generated at the sharp AFM tip, mounted on an active cantilever, which induces local modifications in the substrate material, such as desorption or structural changes. This approach is particularly advantageous for its simplicity and ability to pattern on a wide range of materials [68] without the need for additional processing steps. It is worth highlighting that FEL serves as an alternative to EBL, particularly excelling in low-sensitivity EBL resists. However, FEL has yet to achieve the capability of locally etching or removing a target substrate, as demonstrated by EFLAO. Additionally, most successful FEL patterning experiments have utilized non-commercial probes equipped with integrated piezoresistive read-out and thermomechanical actuation (active cantilevers). Despite these challenges, Behzadirad et al. demonstrated that using sharp gallium nitride nanowires (GaN NWs) as tips enables atomic-scale patterning [67] (<1 nm) on a molecular resist under vacuum conditions.

#### 2.4 Applications of the LAO process

The fact that LAO can be performed with an inexpensive AFM setup makes it invaluable for the prototyping of nanoelectronics devices such as metal–insulator–metal diodes [69] and field-effect transistors (FETs) based on silicon nanowires [42,70–73]. Usually, a narrow oxide mask is patterned on top of the active layer of a silicon-on-insulator (SOI) substrate; further etching removes the excess silicon, leaving a free-standing silicon nanowire matching the shape and size of the original oxide mask. The nanowire is then contacted by micrometer-sized metal contacts by either photolithography or EBL. By a similar approach, it was possible to fabricate nanoscale FETs on thin-layer TMDs [21,24,36]. Graphene-based nanoelectronics devices present a further challenge, single- or few-layer graphene flakes have been preferentially “removed” [23] (as mentioned before describing EFLAO), mechanically cleaved [74], or thermally etched [75] rather than chemically modified [22,35,76–77]. This was done mainly to preserve the electrical characteristics of pristine graphene. The unique capacity of LAO to build ultranarrow dielectric barriers in a 2D fashion has been also exploited to fabricate quantum devices on semiconductors [71,78].

#### 2.5 Materials used in LAO

A diverse range of materials has been utilized in LAO, leveraging their distinct oxidation behaviors, structural characteristics, and applications. This review focuses on metallic, semiconducting, and 2D materials, while excluding significant efforts on organic semiconductors [79] and functionalized substrates [80–82] (like self-assembled monolayers [83]), which fall beyond the scope of this work. Nevertheless, it is worth mentioning that tip-induced electrochemical oxidation has also been demonstrated on organic monolayers, most notably in the seminal work of Sagiv and coworkers. They showed that alkylsilane monolayers can be patterned nondestructively by converting terminal –CH_3_ groups into –COOH functions [84], enabling chemical templating and subsequent selective self-assembly of organic or inorganic species [85]. Follow-up work further demonstrated that such patterned monolayers can even support single-layer ionic conduction along predefined nanoscale paths [86]. These studies extend LAO-type surface modification to molecular films and highlight its relevance beyond inorganic substrates. Table 2 presents a comparative analysis of the key materials that have been successfully utilized for LAO experiments and device fabrication.

**Table 2:** Overview of materials processed by local anodic oxidation and recent experimental outcomes.^a^

Material	Key experimental results	Applications	Min. feature size (lateral)	By-product/oxide	Key challenges

silicon	precise SiO_2_ layer growth with nanometer control	transistors, dielectric isolation layers, and SiNWs	4 nm [50]	SiO_2_	limited features height and 20–80% relative humidity
silicon carbide (SiC)	high oxide thickness with SiO*_x_*C*_y_* composition	high-temperature electronics	50 nm [34]	SiO*_x_*C*_y_*	residual charges and mixed oxides
graphene	generate GO features within a graphene channel of a FET	flexible electronics and quantum devices	20 nm [22]	GO	semiconducting GO features and oxidation confinement
graphene oxide	reduction to graphene nanoribbons with sub-20 nm widths	flexible electronics and quantum devices	65 nm [87]	rGO	controlling reduction and reproducibility
MoS_2_, MoSe_2_, and WSe_2_	plasma-enhanced oxidation	optoelectronics and transistors	sub-10 nm constrictions [21]; 10 nm precision (EFLAO) [23]	MoO_3_ or WO_3_	humidity sensitivity and oxidation confinement
titanium	TiO_2_ layers for optical and catalytic applications	photocatalysis and sensors	n.a. (conventionally sub-µm)	TiO_2_ [88]	sputtered Ti layer and controlling uniformity
aluminum	thin, uniform oxide layers	optical devices and plasmonics	n.a. (conventionally sub-µm)	Al_2_O_3_ [89]	sputtered Al layer and requires strict voltage control
hBN/graphene	no electrode, AC-driven trench patterns with smooth edges	quantum heterostructures	10 nm precision (EFLAO) [23]	none	n.a.

^a^Typical LAO operating conditions: voltage: 5–20 V; humidity: 35–55% RH; tip speed: 0.1–10 µm·s^−1^; pulse duration: 1 ms to 1 s.

**2.5.1 Semiconductors.** Semiconductors like silicon have been widely studied for LAO due to their extensive use in microelectronics, the predictable oxidation behavior, and compatibility with microfabrication processes. The formation of SiO_2_ enables high-resolution patterning, often achieving feature sizes below 10 nm, making it invaluable for applications like insulating layers in transistors. Additionally, the stability and integration of SiO_2_ within semiconductor workflows make it a reliable choice for robust electronic devices. However, silicon’s limitations include dependence on stringent surface preparation, Si crystal orientation (higher reaction rate on the (111) surface than on the other surfaces), and environmental factors, such as humidity, which can impact reproducibility. LAO-grown oxide is less dense than thermally grown oxide, and mechanical stress between the substrate and oxide layer may lead to defects. In addition, the insulating nature of SiO_2_ limits its versatility compared to materials that allow for tunable electronic or structural features. While silicon is ideal for standard electronic applications, its constraints highlight the need for alternative materials in more diverse or multifunctional nanoscale devices. LAO has likewise been demonstrated on III–V semiconductors, most notably GaAs and related heterostructures, where the patterned oxide lines were used as templates for nanoscale device fabrication [90].

**Figure 4:**
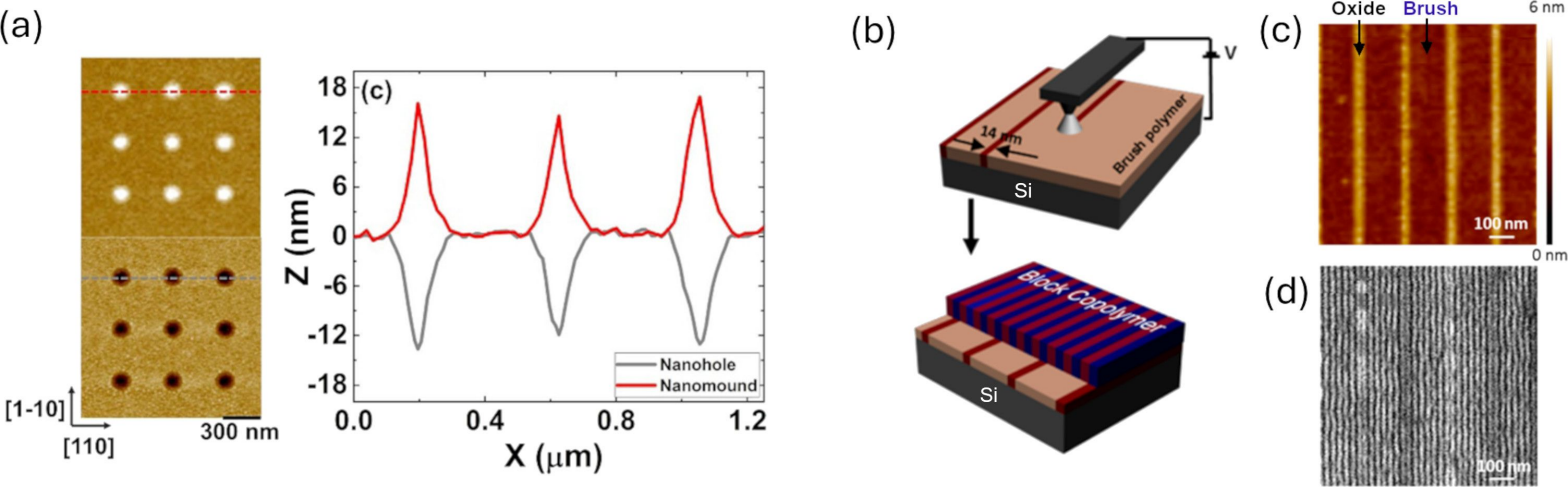
Applications of LAO for nanoscale patterning and directed self-assembly. (a) AFM image of nanodots formed by LAO on an InP substrate and corresponding nanoholes obtained after selective acid etching. Reproduced from [93] (© 2021 C. Ovenden et al., published by IOP Publishing Ltd, distributed under the terms of the Creative Commons Attribution 4.0 International License, https://creativecommons.org/licenses/by/4.0). (b) Schematic illustration of the directed self-assembly process, in which oxidative SPL creates guiding patterns for block copolymers on a Si-functionalized chip. (c) AFM topography showing a representative region of the patterned template. (d) SEM image of the resulting aligned block-copolymer lamellae. Panels (b–d) adapted with permission from [96], Copyright 2014 American Chemical Society. This content is not subject to CC BY 4.0.

Silicon carbide (SiC) is an interesting material for LAO, offering unique advantages over silicon due to its high thermal conductivity, chemical inertness, and suitability for high-temperature environments. The formation of mixed oxides [91] (SiO*_x_*C*_y_*) during LAO provides opportunities for tailoring electrical properties, making SiC valuable for robust microelectronic and power devices. Its self-limiting oxide growth allows for precise control of layer thickness, which is critical for nanoscale patterning. Experimental observations report self-limiting oxide growth exceeding 100 nm [34]. As crystal orientation affects LAO on Si, the oxidation rate on c-plane 4H-SiC is higher than on other planes [92]. Challenges with SiC include the incorporation of residual charges in the oxide layer, which can affect electrical performance. Yet, SiC’s superior durability and unique properties make it a promising material for applications in harsh and demanding environments, though it is less versatile than newer 2D materials or heterostructures. Recently Ovenden et al. demonstrated conventional LAO on InP [93] intending to obtain quantum dot arrays [94]. Nanohole and nano-oxide mound radius and depth were controlled independently by altering tip bias and humidity, with a maximum nanohole depth of ≈15 nm after acid etching. The obtained nanoholes are shown in Figure 4a. The silicon surface can be functionalized and subsequently patterned through local oxidation [95], as illustrated by a method for generating guiding patterns for the directed self-assembly of block copolymers (BCPs) [96]. By integrating chemical and topographic modifications, these guiding patterns efficiently align BCPs into an ordered parallel lamellar arrangement, as depicted in Figure 4b–d.

**2.5.2 Metals.** Metals like titanium and aluminum are frequently utilized for plasmonic devices and nanoscale sensors. Oxidation of titanium during LAO forms TiO_2_ [69,88], a process highly dependent on relative humidity and applied voltage. Patterns on titanium exhibit uniform thickness, making it well-suited for applications in nanoelectronics devices, memristive memory elements, and lithographic masks. Typically, the titanium layer is deposited on semiconducting or insulating substrates, such as SiO_2_, to facilitate effective LAO. A study by Tominov et al. [18] introduced forming-free titanium oxide-based memristive nanostructures created through AFM-assisted local oxidation. This work demonstrates the fabrication of nanoscale resistive switching devices while eliminating the need for the electroforming step, which often requires high voltages that can compromise stability. The study revealed that thinner titanium oxide layers enhance resistive switching performance by providing higher resistance ratios and more uniform switching behavior. Furthermore, compositional analysis showed that TiO_2_ dominates the oxide structure, while deeper regions exhibit increased contributions of Ti_2_O_3_ and TiO. The authors claim that the precise control of oxygen vacancies during LAO would enable the reproducible and scalable production of high-performance ReRAM devices.

Aluminum oxidation by AFM LAO is well suited for forming thin protective layers. The high reflectivity of Al patterns offers applications in optical devices. Successful LAO experiments were performed on a thin Al layer sputtered on SOI substrates [89]. Similarly, oxidation of a 10 nm thick Nb layer was successfully achieved by Heidelberg and colleagues [29].

A more recent study demonstrates the use of LAO to nanostructure thin amorphous vanadium oxide (VO*_x_*) films [97], converting them locally to vanadium pentoxide (V_2_O_5_) for selective removal. Achieving sub-50 nm lateral precision and sub-0.3 nm depths, the process is influenced by humidity, film thickness, and voltage, following the Cabrera–Mott model. This clean, precise approach highlights LAO’s potential for photonic and nanoelectronics applications, aligning with advances in LAO research.

**2.5.3 Two-dimensional materials.** 2D materials are at the forefront of LAO research, owing to their exceptional structural and electronic properties, and have undoubtedly become a major focus for experts in scanning probe lithography. They exhibit oxidation behaviors that differ fundamentally from those of bulk semiconductors and metals. Graphene, TMDs, and their heterostructures each follow distinct reaction pathways under LAO, leading to markedly different feature geometries and device possibilities. LAO can define ultranarrow dielectric barriers, constrictions, and etched channels directly on the active material with nanometer precision and without resist contamination. In 2D materials, this allows for the fabrication of graphene nanoribbons, quantum point contacts, and TMD nanotransistors by locally modulating conductivity or selectively removing material.

**2.5.4 Graphene and graphene oxide.** Interest in graphene peaked in the last decades, with several attempts to couple LAO with single/few-layer graphene flakes [22,98–99], epitaxial graphene [20,100], graphene grown by thermal decomposition of SiC [77], and graphite [101]. By applying local oxidation in a humid environment, the resulting reactive oxygen species, such as OH^−^ and O_2_^•−^, oxidize the graphene surface, forming graphene oxide (GO) or trenches, depending on the applied voltage and process conditions. Key experimental findings highlight that the type and size of features depend on several factors, including the magnitude of the tip voltage, relative humidity, and writing speed [22]. For instance, voltages below a threshold of −5 to −10 V led to the formation of GO. Beyond −16 V, ablation occurs, resulting in the formation of trenches where the carbon atoms are removed [98,102]. Feature dimensions are also affected by the tip speed; slower speeds result in wider features, while faster speeds narrow the oxidized lines. The degree of oxidation increases with higher RH, which enhances the size and reactivity of the water meniscus. The structural and electronic behavior of graphene during LAO is influenced by its layer stacking. As pointed out by Liou and coworkers [20], the inherently 2D nature of these materials influences the outcomes of LAO processes. For instance, oxidation can lead to local protrusions significantly taller than the thickness of a single atomic layer due to the formation of an oxide bump in the conductive substrate beneath the 2D material. When a layered material is placed on an insulating substrate (such as SiO_2_) and contacted from the top, oxidation can disrupt lateral conductivity, effectively isolating the 2D material and leading to unwanted defects. Bernal-stacked graphene allows for uniform oxidation across layers, while non-Bernal stacked graphene limits oxidation to the topmost layer due to reduced electronic interactions between layers. Raman spectroscopy and micro-XPS studies confirm these effects, revealing oxygen incorporation patterns and changes in chemical composition. In summary, the versatility in achieving selective oxidation or ablation expands the potential for device fabrication, including resistive switching elements and nanosensors, highlighting its importance in advanced graphene-based technologies.

As already mentioned, Li et al. [23] developed EFLAO, a novel technique driven by high-frequency AC voltage, enabling precise and high-quality nanoscale patterning of low-dimensional materials without the need for prefabricated microelectrodes. This method overcomes the limitations of conventional LAO, such as dependency on conductive substrates and residue formation. Key contributions include the successful fabrication of graphene nanoribbons with smooth edges and widths as small as 10 nm, demonstrating superior etching quality comparable to natural edges. Figure 3e shows the topography of an ultrathin graphene nanoribbon with width of ≈10 nm and edge roughness of ≈2 nm. As an etching method that operates without the need for an external electrode, once parameters such as AC frequency and voltage are properly optimized, the technique enables the formation of clean, residue-free patterns while maintaining consistent and reliable oxidation. The study also extends to heterostructures, such as graphene/hexagonal boron nitride (hBN) as presented in Figure 3f, achieving uniform etching and demonstrating that heterostructures consisting of two different materials can also be tailored by EFLAO. The approach relies on capacitive coupling through dielectric substrates to generate localized electric fields and supports patterning at varying frequencies, with optimal performance at frequencies above 10 kHz. Additionally, the technique avoids common issues of oxide residues and ensures scalability with high reproducibility. This breakthrough positions EFLAO as a promising tool for next-generation nanolithography, offering flexibility and simplicity for fabricating ultraclean nanodevices.

**Figure 5:**
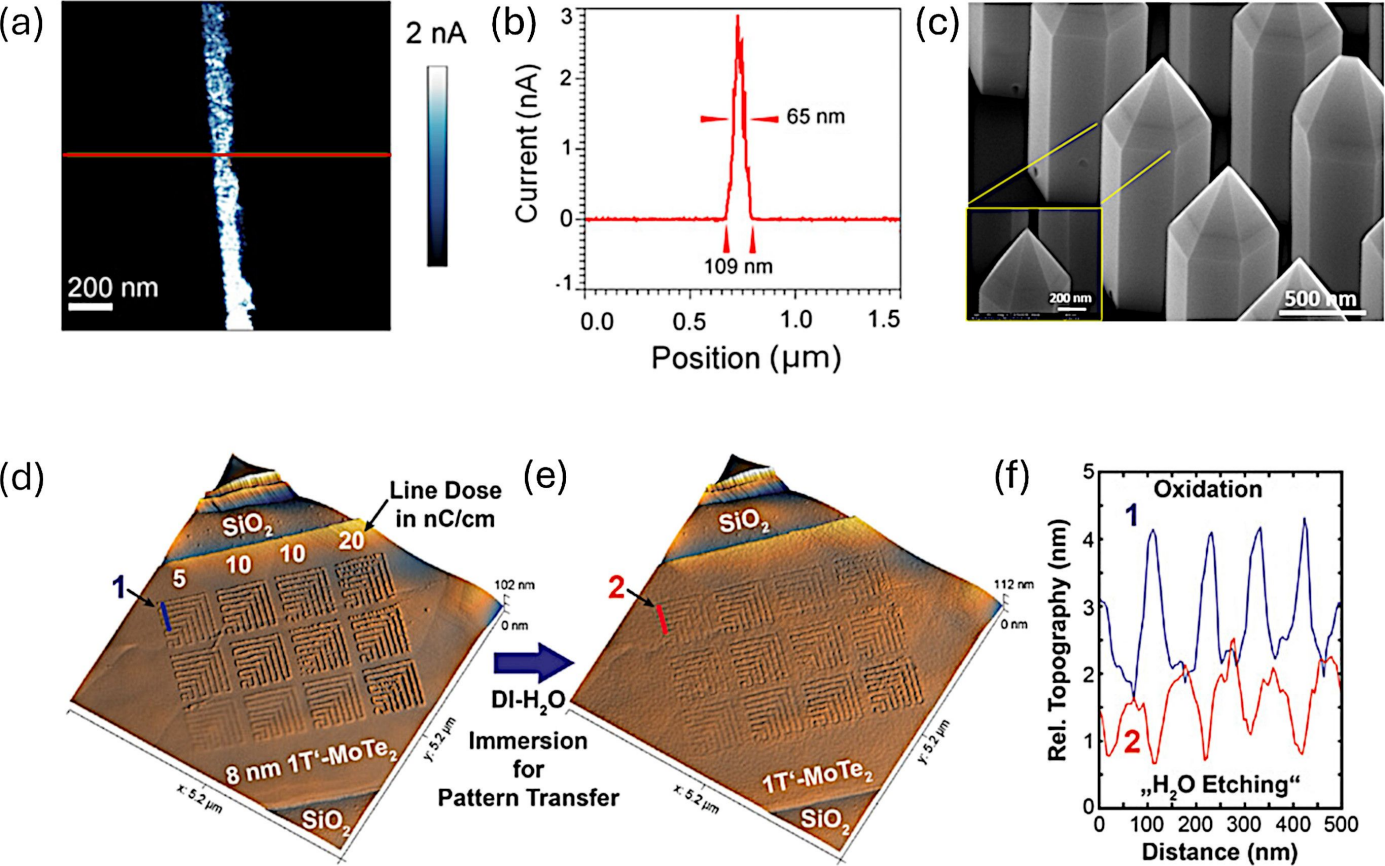
Examples of advanced SPL-based patterning on graphene oxide and MoTe_2_. (a) Conductive-AFM image of a single reduced graphene oxide (rGO) line fabricated by localized electrochemical reduction. (b) Corresponding current profile demonstrating restored conductivity within the nanoscale rGO feature. Panels (a,b) used with permission from [87] (“Nanoscale reduction of graphene oxide thin films and its characterization”, by M. Lorenzoni et al., *Nanotechnology*, Vol. 26, Article No. 285301, published on 29 June 2015; DOI 10.1088/0957-4484/26/28/285301); © 2015 IOP Publishing; permission conveyed through Copyright Clearance Center, Inc.). This content is not subject to CC BY 4.0. (c) Array of GaN nanowires used as ultrasharp, mechanically robust AFM tips for high-resolution SPL. Adapted with permission from [67], Copyright 2021 American Chemical Society. This content is not subject to CC BY 4.0. (d) AFM topography showing nanoscale oxidation patterns created on a 1T′-MoTe_2_ nanoribbon. (e) Resulting surface morphology after selective removal of the oxide in deionized water. (f) Cross-sectional profiles extracted from panels (d) and (e). Panels (d–f) adapted from [36] (© 2023 C. Reuter et al., *Advanced Materials* published by Wiley-VCH GmbH, distributed under the terms of the Creative Commons Attribution 4.0 International License, https://creativecommons.org/licenses/by/4.0).

Reduced graphene oxide (rGO) resembles graphene but with some residual oxygen and structural defects. rGO comprises ordered sp^2^ domains with nanosized dimensions interspersed between highly disordered sp^3^ domains with oxygen-containing functional groups. This indeed limits the film’s conductivity and mobility. Reversing the bias in a standard LAO setup enables the restoration of conductivity in GO layers or flakes through a localized reduction reaction [87,103] or a localized catalytic reaction [104]. In Figure 5a,b, we show a conductive AFM image of a single restored rGO feature [87] obtained after local reduction by SPL, with a lateral size below 100 nm. Even better results on GO, in terms of lateral resolution, have been achieved by thermochemical nanolithography [105]. The electrical properties of rGO patterns obtained in both ways (electrochemically and thermochemically) are excellent, with rGO presenting a bulk conductivity that is comparable to the values reported for chemically reduced GO (>10^4^ S·m^−1^).

**Figure 6:**
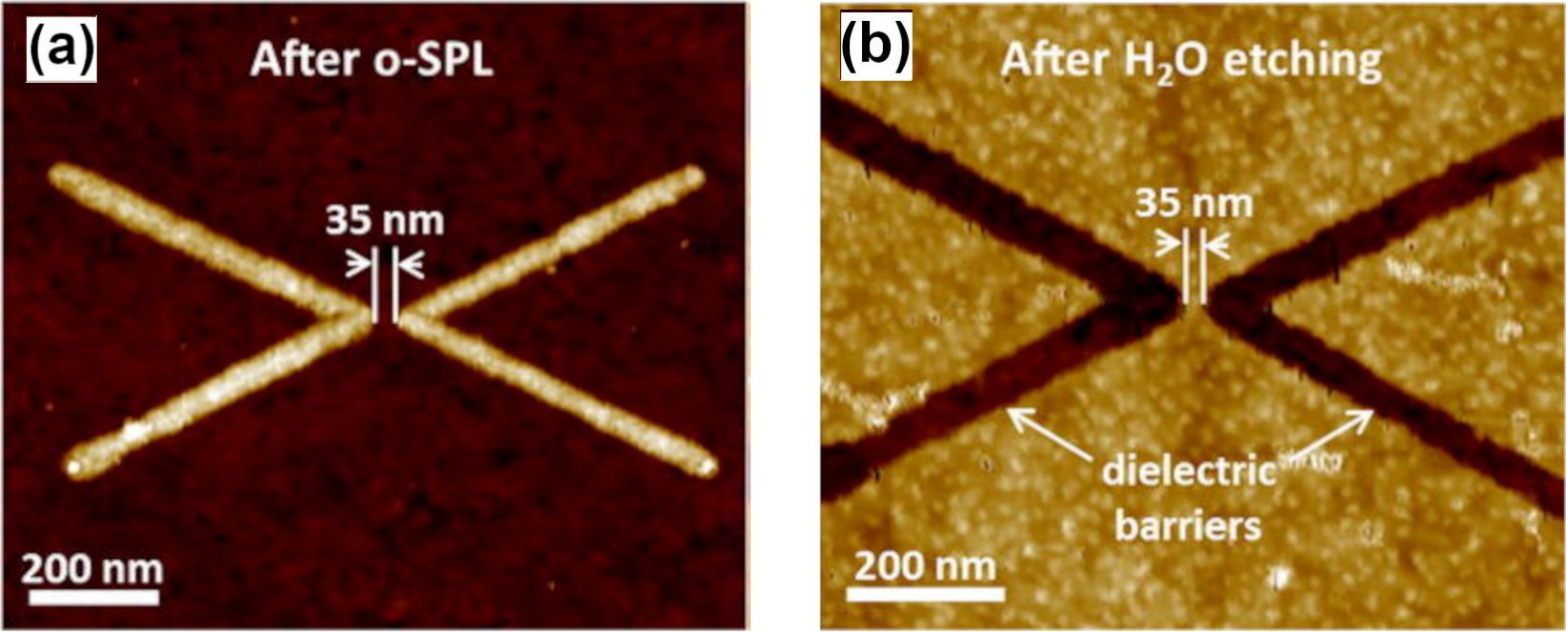
High-resolution patterning of transition metal dichalcogenides by LAO. (a) AFM topography of a ≈35 nm constriction patterned on few-layer WSe_2_ prior to etching. (b) AFM topography of the same constriction after selective dissolution of the oxide in deionized water, revealing the etched trench. Panels (a,b) adapted from [24] (© 2016 A. I. Dago et al., published by AIP Publishing, distributed under the terms of the Creative Commons Attribution 4.0 International License, https://creativecommons.org/licenses/by/4.0).

**2.5.5 Transition metal dichalcogenides.** TMDs, such as MoS_2_, MoSe_2_, WSe_2_, and MoTe_2_, have emerged as highly promising materials for LAO due to their unique electronic, structural, and chemical properties. LAO on TMDs leverages their atomically thin layers and tunable band structures to create high-resolution nanoscale features, including nanoribbons, trenches, and transistor channels. The oxidation process typically involves converting the metal component of TMDs into higher oxidation states, such as MoO_3_ or WO_3_, which can be selectively etched or retained depending on the desired application. Studies have demonstrated that LAO can achieve sub-20 nm patterning resolution on MoS_2_ [21] and WSe_2_ [24], enabling the fabrication of nanoribbon-based FETs. Oxygen plasma-assisted LAO has been employed to enhance the precision and reduce defects in the resulting patterns [21], making the process highly suitable for device applications requiring nanoscale control. In fact, LAO patterning of both MoS_2_ and WSe_2_ is enhanced by an initial oxygen plasma treatment, which forms a uniform oxide layer of approximately 3 to 4 nm on the surface of the flake. This pre-treatment not only reduces the flake thickness but also minimizes the impact of the underlying dichalcogenide crystal lattice on the quality of the LAO patterns, surpassing previously reported results [26]. One example of such pattering capabilities is reported in Figure 6 showing a 35 nm constriction fabricated on few-layer WSe_2_, before and after etching in water.

Additionally, the environmental sensitivity of TMDs, such as the anisotropic oxidation of 1T′ MoTe_2_ under humid conditions, has been exploited to achieve unique etching profiles and selective modifications. In a study by Reuter et al., low-energy electrons emitted from the tip have been used to induce nanoscale oxidation on a MoTe_2_ nanosheet surface under ambient conditions [36]. The study demonstrates a novel, resist-free method for nanoscale patterning of MoTe_2_ in both its semiconducting (2H) and semi-metallic (1T′) phases using a tip-based lithographic approach; examples of such patterns are shown in Figure 5d–f. The method does not induce measurable phase transitions, preserving the intrinsic material properties and is, therefore, suitable for exploring fundamental behaviors of MoTe_2_ at the nanoscale. Interestingly, increasing either the relative humidity or the line dose promotes greater oxide formation, resulting in deeper structuring of MoTe_2_ after oxide removal (i.e., etching depths of up to 6 nm have been observed following water etching). In contrast, raising the bias voltage primarily broadens the lateral growth of the oxide without significantly impacting its height. The authors demonstrated that the hybrid approach (combining electron emission with oxidation) effectively addresses common limitations observed in 2D samples contacted via a top electrode. By regulating current peaks and operating within a controlled field-emission regime, this method suppresses uncontrolled oxidation and minimizes the formation of line defects. The findings suggest broader applicability of such techniques to other tellurium-containing TMDs, emphasizing scalability and environmental considerations. This precise control has significant implications for applications in photonics, nanoelectronics, and quantum computing. However, challenges remain, including the influence of humidity on patterning reproducibility and the need to mitigate structural degradation during the oxidation process. Transitioning to t-SPL would ultimately enable operation in a dry nitrogen environment, thereby preventing oxidation, a capability recently demonstrated by Liu and coworkers [106].

It is to mention that also Shilov and coworkers focused on electrode-free nanopatterning of thick multilayer van der Waals (vdW) heterostructures [64]. This approach, which relies on applying a low-frequency (10 kHz) AC voltage between the AFM tip in contact mode and the grounded substrate, achieves high-resolution etching (down to ≈100 nm) without the need for a humid environment. The technique demonstrates its effectiveness across a wide range of heterostructures, including those composed of hBN, graphene, WSe_2_, and NbSe_2_, with patterns such as trenches, constrictions, and dots created at nanoscale precision. The method capitalizes on electric field enhancement, facilitated by assembling vdW heterostructures on conductive graphite slabs, which localize and amplify the electric field. This enhancement enables the breakdown of dielectric materials, such as hBN, initiating chemical reactions that drive the etching process. Notably, the method is robust under low humidity (RH ≈ 20%). This study emphasizes precise control, reproducibility, and compatibility with complex vdW heterostructures, similar to the previously discussed work on EFLAO, while also asserting the advantage of functioning effectively in a less humid environment.

### 3 Remaining challenges and perspectives

This section briefly highlights future directions to address current limitations and expand the scope of LAO.

#### 3.1 Novel materials

While semiconductors, metals, and 2D materials have been extensively studied, LAO should be further explored for materials like black phosphorus (BP) and 2D transition metal carbides (MXenes) that may introduce new oxidation dynamics. Regrettably, key factors such as oxygen, humidity, and light, which contribute to the ambient degradation of few-layer BP [107], may present significant challenges for the application of LAO. The conductive properties of MXenes range from metallic to semiconductor-like [108], making them promising candidates for LAO experiments (see Section 3.2.3). Delaminated individual flakes, reaching lateral sizes of up to 40 μm [109], are already available and can be utilized for patterning multiple contacts and fabricating FETs, like earlier approaches with single graphene flakes. However, their susceptibility to degradation in the presence of water and oxygen remains a significant challenge [110]. Yet, for both materials, an ultrathin casing layer of oxide or polymer resist might not represent an obstacle to LAO processing, as already demonstrated [41,63].

Polymers remain an underexploited material domain for LAO experiments. Suitable polymeric materials with electrochemical patterning capabilities are rare. With few exceptions, like the organic semiconductor [111] or organometallic multilayers [112], polymers, with their markedly insulating nature, tend to pose serious challenges to current-driven local oxidation by the scanning probe. Nevertheless, it is indeed possible to cross-link a thin molecular resist layer by LAO to induce local cross-linking [113].

Expanding LAO to organic and biocompatible materials holds significant potential for creating functional surfaces tailored for medical applications [114]. By leveraging LAO’s precise patterning capabilities, researchers can develop surfaces with nanoscale features that promote specific interactions with biological systems. For instance, functionalized surfaces could enhance cell adhesion, guide tissue growth, or enable selective biomolecule immobilization. As an example, Martínez et al. [115] combined top-down oxidative nanolithography techniques and bottom-up electrostatic interactions to pattern single molecules of ferritin on silicon surfaces. Furthermore, biocompatible materials such as polymers can be selectively oxidized to create hydrophilic regions or chemically reactive sites, enabling controlled interactions with proteins, cells, or other biomolecules. However, challenges remain in ensuring that the LAO process does not degrade the material’s biocompatibility or structural integrity, particularly for sensitive organic substrates.

#### 3.2 Enhancing process precision

**3.2.1 Advanced tip design.** Developing tips with improved durability and conductivity (e.g., multilayer coatings or adaptive tip shapes) could mitigate wear and maintain precision over extended use. Selecting an appropriate tip material is crucial for achieving high image and pattern resolution during LAO. A suitable tip must meet three key criteria: (i) high electrical conductivity, (ii) strong mechanical stability (durability), and (iii) an optimal geometry characterized by a sharp apex and appropriate cone angle. Traditional materials used in LAO experiments are highly doped Si or metals including W, Pt, Au, and Ir. Si-based tips are cheap but lack mechanical durability, frequently suffering from deformation or damage during imaging and patterning. Metal-coated tips all offer excellent electrical conductivity and improved mechanical stability. However, these coatings can degrade over time due to mechanical wear and electrochemical reactions, especially under high contact forces (1000–1500 nN) and elevated bias voltages (10–25 V). This degradation can lead to tip blunting or delamination, reducing resolution and altering oxide feature dimensions. Recently, GaN NWs have demonstrated exceptional mechanical properties [67], making them promising candidates for SPL-related applications (Figure 5c). Diamond carbon-coated tips offer exceptional mechanical properties but are limited by their relatively large minimum tip radius. Solid platinum silicide (PtSi) tips provide a strong alternative [116], combining durability with consistent conductivity, even when the external coating peels off.

**3.2.2 Substrate surface engineering.** The substrate plays a critical role in determining the precision and quality of features produced by LAO. Pre-treatment techniques, such as oxygen plasma cleaning, ensure that the substrate surface is free of contaminants and uniform, providing a consistent environment for the oxidation reaction. Similarly, applying thin surface coatings, like hydrophilic layers, can enhance the formation and stability of the water meniscus, which is crucial for consistent oxidation. Engineering the substrate’s surface energy is another promising avenue. Substrates with controlled hydrophilic or hydrophobic properties can help localize the oxidation process, reducing lateral spreading and improving feature sharpness. Advanced techniques, such as ALD to create ultrasmooth and defect-free surfaces, further enhance the precision and reproducibility of LAO.

**3.2.3 Material-specific approaches.** Tailoring LAO parameters to the specific properties of the material being patterned is essential for achieving high-resolution and uniform features. Different materials exhibit varying oxidation kinetics, conductivity, and susceptibility to environmental conditions. For example, TMDs such as MoS_2_ and WSe_2_ are especially sensitive to phonon scattering, which significantly influences their thermal conductivity, electrical transport properties, and carrier mobility. As a result, precise control over oxidation depth and lateral diffusion during processing may require the use of lower voltages and carefully optimized humidity conditions. Hybrid approaches, which combine LAO with complementary techniques such as chemical vapor deposition or thermal oxidation, can further improve feature resolution and reduce defects. In the context of biocompatible materials, the direct application of LAO for surface oxidation or modification on biological substrates or tissues remains largely unexplored. A promising strategy could involve the patterning of poly-ʟ-lysine (PLL) thin films, a well-established material in biotechnology [117]. Following LAO patterning, protein-resistant polymers like polyethylene glycol can be selectively grafted onto the patterned areas, forming a copolymer with PLL that faithfully replicates the design. This approach has potential for advancing protein patterning, tissue engineering, biomedical implants, and biosensor development.

LAO offers a promising route for fabricating superconducting quantum devices by enabling direct, nanoscale patterning of Josephson junctions without conventional lithography. Using a biased AFM tip, ultrathin oxide barriers can be written locally on superconducting films such as Nb [29,118], TiN [119], or Al, transforming selected regions into insulating junctions that act as weak links for Cooper pair tunneling. This technique allows for the precise creation of Josephson junctions or SQUID loops with tunable critical currents, minimal contamination, and sub-10 nm spatial control. Extending LAO to 2D superconductors like NbSe_2_ [120] or Mo_2_C [121] could further enable atomically clean, reconfigurable qubit architectures, merging nanolithography and quantum engineering into a single scalable process.

**3.2.4 Multitip and parallelized systems.** Scaling and automation, such as parallelized LAO, have been proposed as key solutions to compete with standard optical lithography processes. Multitip AFM setups or arrays could enable scalable fabrication while preserving nanoscale resolution. Currently, thermal SPL technologies utilizing active cantilevers appear more likely to advance toward parallel operations [122–123], as illustrated by the recently introduced Decapede system from Heidelberg Instruments, a commercial multicantilever NanoFrazor platform that demonstrates scalable parallel t-SPL. By contrast, LAO remains primarily a prototyping technique best suited for laboratory-scale setups. It is worth noting that recently developed cantilever-free scanning probe technologies [124] address the scalability challenge by employing large arrays of polymer pens, effectively overcoming the inherently low throughput of traditional SPL.

**3.2.5 Machine learning.** Optimizing LAO process parameters (such as pulse amplitude, width, and tip–sample interaction) remains a complex challenge. Recent developments in numerical simulation and machine learning (ML) offer promising avenues to address these complexities and might reduce dependency on trial-and-error methodologies. For example, numerical simulations can be employed to explore complex reaction mechanisms involved in nanomanufacturing processes at the nanoscale. This approach has recently been used to clarify the anisotropic effects observed during the LAO process on silicon [125]. Supervised learning models can be trained on experimental data to predict feature geometries, enabling more precise control over the oxidation process. Additionally, reinforcement learning approaches can facilitate real-time adaptive control of pulse parameters to achieve desired nanostructures. Notably, a study by Zhang et al. [126] introduced a semantic segmentation framework that automates the evaluation of nanolithography results, significantly accelerating the optimization of process conditions and parameters. By integrating such ML-based frameworks, LAO nanolithography can transition from empirical methodologies to more deterministic and efficient fabrication processes, paving the way for advancements in nanoelectronics and photonics.

## Conclusion

The continued development of LAO will rely on interdisciplinary efforts to innovate materials, processes, and applications. Advances in tip design, dynamic operation modes, and environmental control have significantly enhanced its precision and reproducibility, while substrate engineering and material-specific approaches might expand its applicability to complex systems like TMDs, other 2D layered materials and biocompatible surfaces. Despite challenges like tip wear and environmental sensitivity, innovations such as electrode-free local anodic oxidation are extending the potential of LAO toward scalable and industrially viable solutions. By addressing these challenges and leveraging its strengths, LAO could eventually become a cornerstone technique in next-generation nanofabrication.

## Data Availability

Data sharing is not applicable as no new data was generated or analyzed in this study.
